# Trend Analysis of the Sex Ratio at Birth in Greece

**DOI:** 10.7759/cureus.80098

**Published:** 2025-03-05

**Authors:** Nikolaos Vlachadis, Chryssi Christodoulaki, Nikolaos Machairiotis, Konstantinos Louis, Periklis Panagopoulos

**Affiliations:** 1 Department of Obstetrics and Gynecology, General Hospital of Messinia, Kalamata, GRC; 2 Third Department of Obstetrics and Gynecology, National and Kapodistrian University of Athens, Medical School, Attiko Hospital, Athens, GRC; 3 Family Planning Unit, Third Department of Obstetrics and Gynecology, National and Kapodistrian University of Athens, Medical School, Attiko Hospital, Athens, GRC

**Keywords:** birth, greece, human reproduction, sex ratio, trend analysis

## Abstract

Introduction: The sex ratio at birth, defined as the ratio of male to female live births, is a key indicator in the study of human reproduction and demographic patterns. This study aims to systematically examine the sex ratio in Greece and analyze its temporal trends over the period from 1956 to 2023.

Materials and methods: Data on the total number of live births in Greece, categorized by sex, were obtained from the Hellenic Statistical Authority based on birth certificate records. The sex ratio for each year was calculated as the number of male births per 1,000 female births. Trend analysis was conducted using joinpoint regression and supplemented with linear regression. The annual percent change (APC) and the beta coefficient were computed, along with 95% confidence intervals (95% CIs).

Results: Between 1956 and 2023, a total of 8,215,934 live births were recorded in Greece. The overall sex ratio at birth across all years was 1,068 male births per 1,000 female births, resulting in 269,468 excess male neonates. The gender ratio at birth ranged from a low of 1,047 in 1996 to a high of 1,085 in 2001. In all reported years, the sex ratio at birth was above 1,050, except for 1996. In the most recent year, 2023, the male-to-female ratio at birth was the second lowest of the entire period, at 1,051. Trend analysis revealed that the sex ratio at birth remained stable from 1956 to 1982 (p = 0.862) and showed a statistically significant downward trend from 1982 to 2023, with an annual percent change of -0.0274 (p = 0.004), or an average annual decrease of -0.297 (95% CI: -0.504 to -0.090, p = 0.006) male births per 1,000 female births.

Conclusions: The sex ratio at birth in Greece has significantly declined over the past four decades, reflecting similar trends observed in other developed nations. Further research is required to investigate the complex biological, demographic, and social factors influencing these trends, providing deeper insight into key aspects of human reproduction.

## Introduction

The sex ratio at birth is defined as the ratio of male to female newborns and serves as a central indicator in the study of human reproduction and demography. It reflects the natural balance between male and female births and is a key measure of population health. Deviations in this ratio can be linked to physiological, genetic, or environmental factors that influence population dynamics. This measure is often referred to as the secondary sex ratio, distinguishing it from the primary sex ratio, which pertains to the sex ratio at fertilization. Understanding the sex ratio at birth provides valuable insights into the reproductive patterns and broader demographic trends within a population. The examination of sex ratios has intrigued scientists for nearly four centuries, and early research established that this ratio is relatively stable and tends to be biased toward males. As early as the 17th century, Graunt observed that the number of male births exceeded that of female births, establishing a sex ratio of 14:13 for the population of London. This predominance of male births was later confirmed by additional researchers. Today, it is well documented that the sex ratio consistently favors males, with an established male proportion of 51.5%, corresponding to a sex ratio value of 1.06 [1-3].

The sex ratio is a critical factor influencing the demographic trends of a population. A skewed sex ratio is a primary reason for establishing the replacement fertility threshold at 2.1 in developed countries, where infant, child, and young adult mortality rates are negligible. Additionally, a higher sex ratio can result in lower growth potential for a population, and conversely, a more balanced sex ratio may facilitate greater growth potential [3].

The sex ratio at birth is a topic of interest in the fields of epidemiology and reproductive medicine. Research has shown that it is influenced by a complex interplay of genetic, biological, environmental, and social factors, with cultural preferences and socioeconomic conditions also playing a significant role [1-3]. Data on the male-to-female ratio at birth are systematically collected in many countries worldwide, and temporal trends in this ratio are monitored to understand the changes and factors that influence it. In the last few decades of the 20th century, a consistent decline in the sex ratio has been observed in many developed countries in Europe and North America [4-6]. The present study aims to systematically present the sex ratio in Greece and analyze its temporal trends from 1956 to 2023.

## Materials and methods

Study population and demographics

The data for this analysis were derived from the total number of live births in Greece, categorized by sex (male or female), over the period from 1956 to 2023. The data were obtained from the Hellenic Statistical Authority [7], based on birth certificate records. A total of 8,215,934 live births were recorded, including both male and female births.

Inclusion and exclusion criteria

All live births registered in Greece from 1956 to 2023, with data categorized by sex, were included in the analysis. Of the total 8,215,934 live births, 1,696 (0.02%) births had the sex unreported. These unreported births occurred exclusively between 1956 and 1962 and were proportionally distributed between the sexes for analysis purposes.

Sample size calculation

The sample size was based on the total number of live births in Greece, which amounted to 8,215,934, with sex data available for 8,214,238 births.

Study parameters

The primary study parameter was the sex ratio, defined as the ratio of male to female live births, multiplied by 1,000, calculated for each year from 1956 to 2023.

Statistical analysis

Trend analysis was performed using the Joinpoint Regression Program version 5.2.0 (National Cancer Institute, Bethesda, MD), which identifies joinpoints, the specific years where significant changes in trends occur. The analysis computed the annual percent change (APC) for each segment between two joinpoints, allowing for up to six segments. Additionally, trends in the sex ratio were assessed using a linear regression model, with the sex ratio as the dependent variable and year as the independent variable. The beta coefficient was calculated to assess the trend over time. Data analysis was performed using SPSS Statistics version 22 (IBM Corp., Armonk, NY) and Microsoft Excel version 2010 (Microsoft Corp., Redmond, WA). A p-value of less than 0.05 was considered statistically significant, and 95% confidence intervals (95% CIs) were also evaluated.

## Results

**Figure 1:**
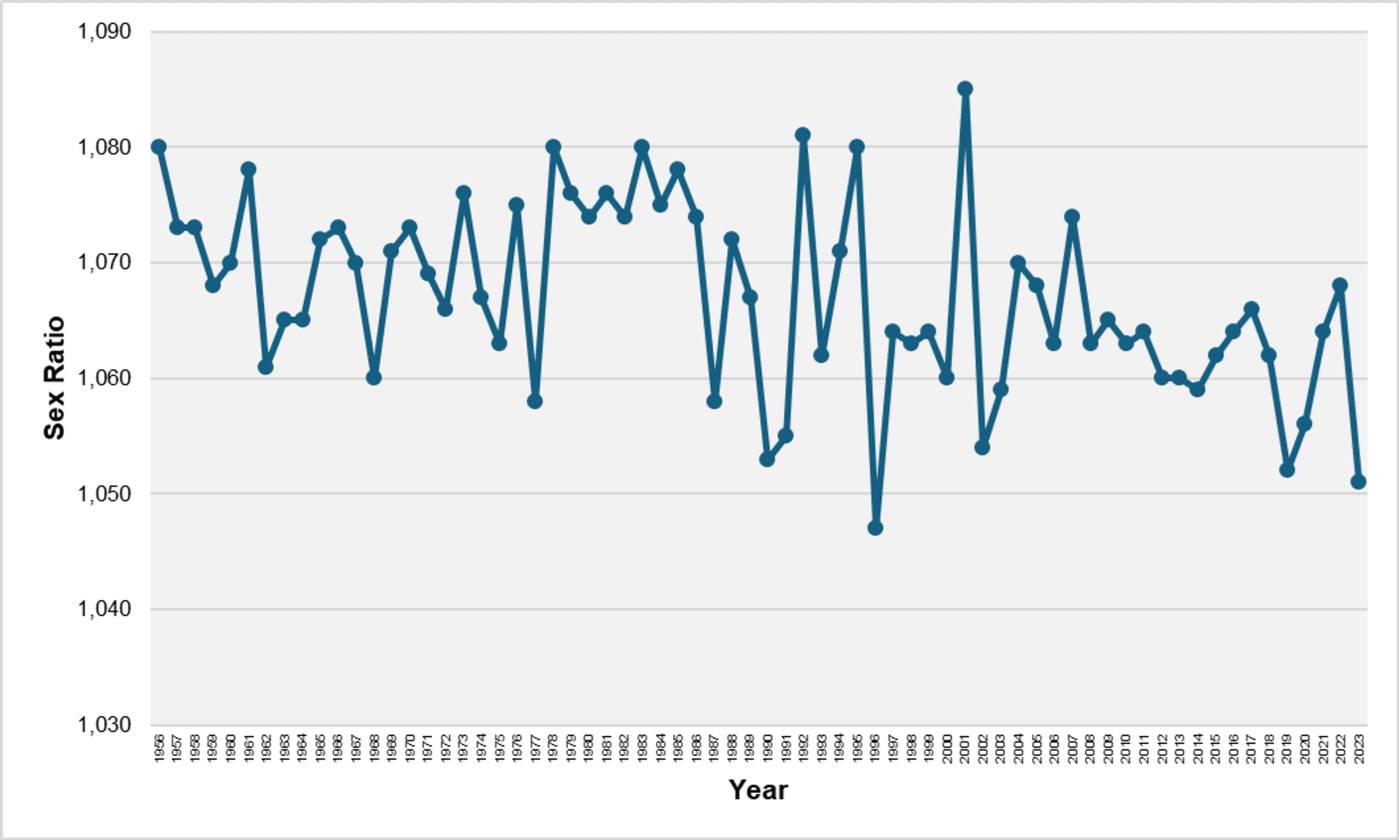
Sex ratio at birth in Greece, 1956-2023 Sex ratio at birth is the number of male births per 1,000 female births.

**Figure 2:**
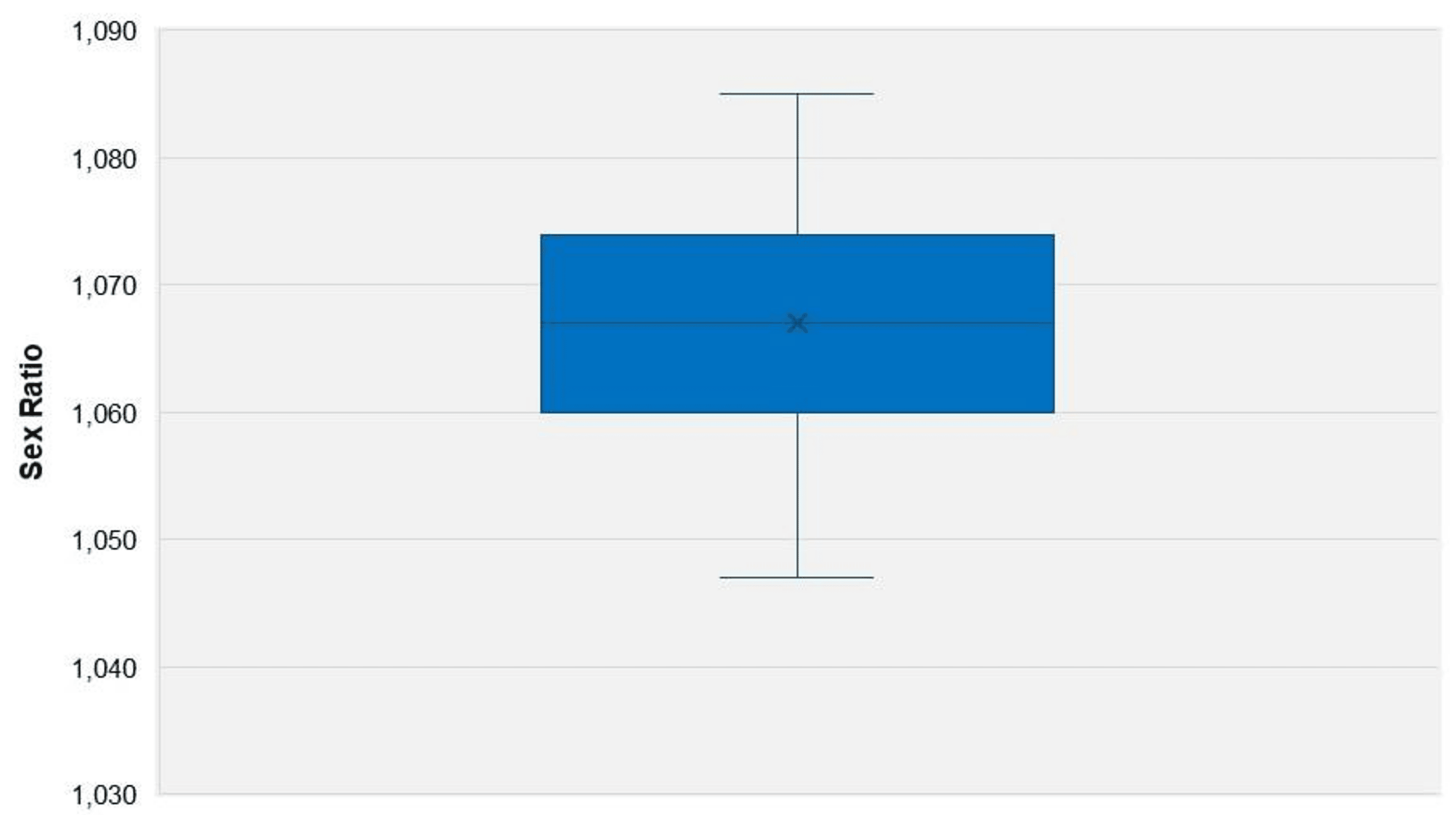
Boxplot of the sex ratio at birth in Greece, 1956-2023 Sex ratio at birth is the number of male newborns per 1,000 female newborns.

Between 1956 and 2023, a total of 8,215,934 live births were recorded in Greece, with 4,242,701 (51.64%) being males and 3,973,233 (48.36%) females. Over the 1956-2023 period, the total number of excess male births was 269,468. The overall sex ratio across all years was 1,068 male births per 1,000 female births, with values ranging from a low of 1,047 in 1996 (males: 51.14%, females: 48.86%) to a high of 1,085 in 2001 (males: 52.04%, females: 47.96%). The median sex ratio was 1,067, and the interquartile range spanned from 1,062 to 1,074. In all reported years, the sex ratio was above 1,050, except for the year 1996. In the most recent year, 2023, the sex ratio was the second lowest of the entire period, at 1,051 (Figure 1, 2).

**Figure 3:**
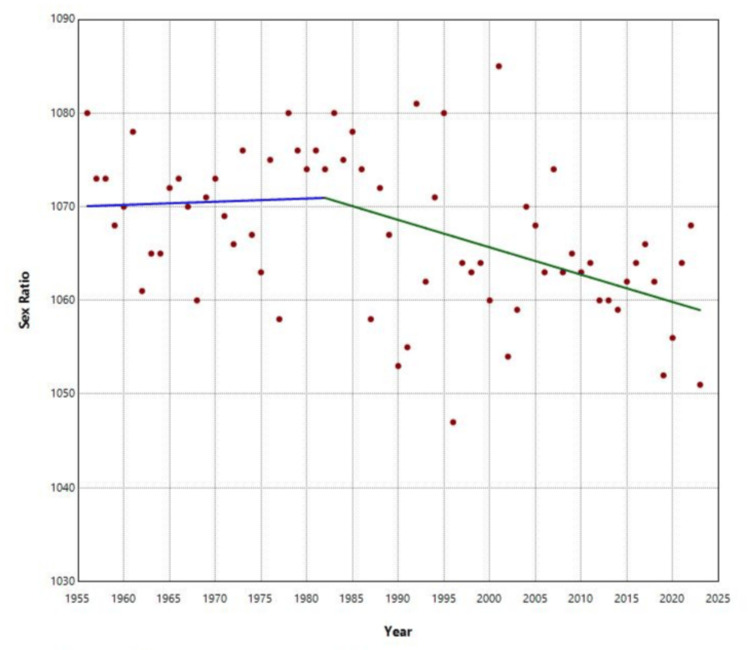
Trends in the sex ratio at birth in Greece, 1956-2023

Trend analysis revealed that the sex ratio remained stable from 1956 to 1982 (APC = 0.0032, p = 0.862). However, during the period from 1982 to 2023, the sex ratio showed a statistically significant downward trend, with an APC of -0.0274 (p = 0.004) (Figure 3).

**Figure 4:**
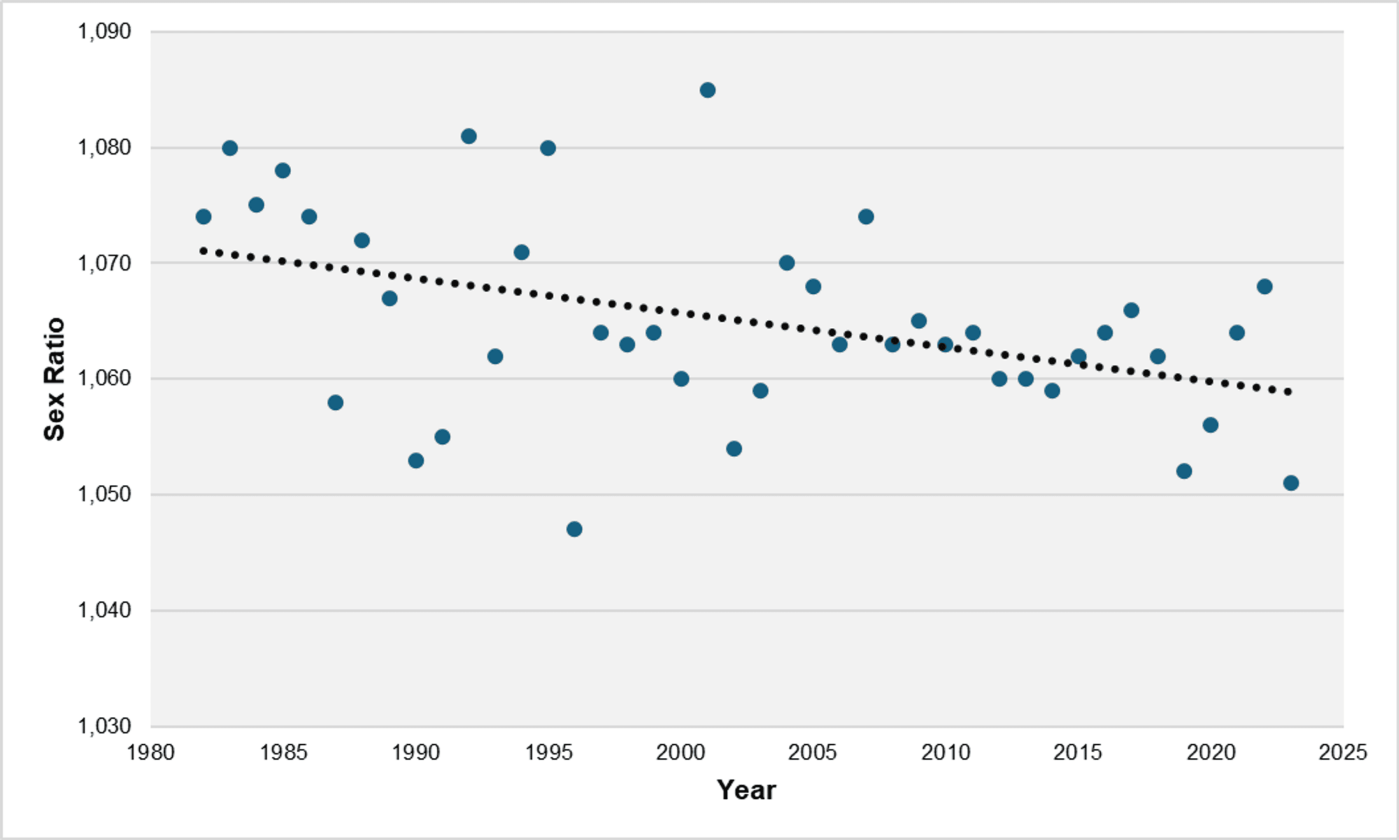
Linear regression analysis of trends in the sex ratio at birth in Greece, 1982-2023

The linear regression equation describing the trend in the sex ratio from 1982 to 2023 was Ŷ = 1,660.9 - 0.297X. The coefficient b = -0.297 (95% CI: -0.504 to -0.090, p = 0.006) indicated an average decrease of 0.297 male births per 1,000 female births per year in the sex ratio (Figure 4).

## Discussion

This study provides a comprehensive overview of the sex ratio at birth in Greece from 1956 to 2023, revealing significant variations and trends over time within the population. The sex ratio at birth in the country ranged from 1,047 to 1,085 male births per 1,000 female births, with an overall average of 1,068 male births per 1,000 female births. This predominance of male births resulted in a total excess of 269,468 boys over the entire period of 68 years. From 1956 to 1982, the sex ratio remained relatively stable, showing a non-significant upward trend. However, during the subsequent period from 1982 to 2023, a statistically significant decline in the sex ratio was observed, with an APC of -0.027%, as determined by the exponential model, or an average reduction of 0.297 male births per 1,000 female births each year, as indicated by the linear model. This declining trend resulted in a sex ratio of 1,051 in 2023, marking the second-lowest value observed during the study period.

The declining trend observed in Greece over the past four decades aligns with patterns seen in other developed nations, including parts of Europe and North America, where sex ratios have also shifted downward in recent decades [8]. For instance, a steady relative decline in male births was documented in the Netherlands from 1954 to 1994 [9] and in Denmark from 1951 to 1995 [10]. While these trends lack a clear explanation, it has been proposed that they may reflect improved living standards and a reduction in fetal loss [8]. In the United States, a statistically significant decline in the male-to-female ratio was observed during the last three decades of the 20th century, with an APC of -0.02%. However, this trend varied by race: the decline primarily affected white populations, while the male-to-female ratio increased among black populations during the same period [3,11]. This racial difference in gender ratio has been attributed to hormonal variations between white and black women, particularly differences in estradiol and testosterone levels [11].

Despite extensive research, the mechanisms underlying the formation of the human sex ratio remain unclear, although it is likely influenced to some extent by factors such as maternal age, race, and birth order. The male-to-female ratio is also shaped by various factors from conception to birth, including pregnancy loss. Notably, the gender ratio among fetuses lost during pregnancy is significantly higher than that among live births. Lower male birth rates have also been linked to additional parameters, such as low maternal weight, stress, and exposure to environmental endocrine-disrupting chemicals [3]. Numerous studies suggest that an elevated secondary sex ratio may be associated with higher socioeconomic and educational levels, while poor nutrition, psychological stress, and socioeconomic exclusion are likely associated with a lower gender ratio at birth [12]. The classical interpretation of the male birth advantage suggests that it is due to increased fertilization of gametes carrying the Y chromosome. According to this view, many more male embryos are formed immediately after fertilization, but they experience higher intrauterine mortality, as indicated by relevant epidemiological data [13,14]. Alternatively, another perspective posits that the gender ratio at conception is 50%-50% and that the higher number of male births results from the decreased mortality of female fetuses at various stages of pregnancy [15].

While the predominance of males over females at birth is observed in nearly all populations worldwide, the degree of this excess varies considerably [16]. For instance, sub-Saharan African countries typically exhibit low sex ratios (values of 1,040 males per 1,000 females or below), while several Asian countries record higher values, often exceeding 1,070 males per 1,000 females [5,14]. One study found a correlation with latitude, showing that boys are more predominant in the southern regions of Europe than in the northern regions, while an inverse correlation was observed in North America. Notably, Greece had the highest proportion of male births among the 26 countries examined in this study [17].

Previous studies on the Greek population have examined trends in the sex ratio and its association with various factors. One study found a statistically significant decrease in the proportion of males at birth over two distinct periods in Greece: 51.72% during 1956-1985 compared to 51.58% during 1986-2005 (p = 0.013). This study also identified a significantly lower sex ratio among children whose fathers were shipyard workers, suggesting that the downward trend in the male-to-female ratio in Greece could be linked to paternal occupational exposure [18]. Additionally, a prospective study in Greece reported a statistically significant association between sex ratio and maternal smoking during pregnancy, with the effect varying by parity [19]. In a study analyzing birth data in Greece from 2004 to 2011, a notably high gender ratio at birth was observed among immigrant mothers, particularly those from Asia, as well as among mothers with low levels of education. The authors interpreted these findings as evidence of sex selection practices within these populations in Greece [20]. Similar results were reported in another national survey using 2006 data, which identified a significantly higher sex ratio of male births among migrant mothers, especially those from Asian countries and Albania [21]. Consistent with our findings, another analysis of national vital data from 1960 to 2006 reported a statistically significant declining trend in the ratio of male to female neonates. This trend did not appear to be influenced by demographic factors such as maternal age or parity. However, the study identified a lower male-to-female ratio in urban areas compared to rural areas after 1980, which the authors attributed to environmental factors potentially disrupting endocrine homeostasis [22].

Several interpretations could explain the declining trends in the sex ratio in Greece after 1980. First, significant changes in population fertility began in the 1980s, marked by declining birth rates and a substantial increase in maternal age [23,24]. Another potential factor is the influence of socioeconomic conditions, particularly over the past 15 years. Greece has faced significant economic fluctuations, including the 2008 financial crisis and subsequent austerity measures, which may have indirectly affected fertility and the gender ratio at birth. Additionally, the past four decades have been marked by a significant increase in multiple birth rates, driven by factors such as advanced maternal age and the growing use of assisted reproductive technologies [25]. This upward trend has likely contributed to the observed decline in the sex ratio, as multiple pregnancies are consistently associated with lower male-to-female ratios [26,27]. Finally, the declining influence of sex selection practices that historically favored male offspring in Greece should be considered. Notably, the sex ratio in Greece was higher than that of 28 other countries during the 1950s and 1970s [4].

The sex ratio at birth in Greece remained relatively high and stable until the early 1980s, after which a significant downward trend has been observed over the past four decades. The strengths of this study lie in its analysis of over eight million births, covering the longest period and the largest number of births compared to previous studies in the Greek population, with data considered valid as they are derived from official birth certificates. Despite the robust dataset and long-term perspective, the study has limitations, as it focused solely on analyzing annual secondary sex ratios for the entire population without exploring potential associations with specific demographic or biological variables. Understanding the causes of this trend is critical, as they may reflect shifts in reproductive behaviors or broader societal changes, with potential implications for fertility policies in Greece.

## Conclusions

The results of this study reveal that the sex ratio at birth in Greece has been relatively high, with a significant downward trend observed since the early 1980s. This decline mirrors trends seen in other developed nations, suggesting that common underlying factors, such as improved living standards, reduced fetal loss, and shifts in reproductive behaviors, may be contributing to the trend. The shrinking birth rates, advanced maternal age, increased use of assisted reproductive technologies, and the decreasing reliance on sex selection practices that historically favored male offspring may have also influenced the recent secondary sex ratio trends in Greece. Further research is needed to explore the complex factors shaping the sex ratio at birth, including biological, demographic, environmental, and cultural influences, which will enhance our understanding of human reproduction.
